# Using cocreated visually informed community mental health education in low‐ and middle‐income countries: A case study of youth substance misuse in Assam, India

**DOI:** 10.1111/hex.13550

**Published:** 2022-06-18

**Authors:** Raginie Duara, Diptarup Chowdhury, Ratul Dey, Sangeeta Goswami, Anna Madill

**Affiliations:** ^1^ School of Psychology University of Leeds Leeds UK; ^2^ Lokopriya Gopinath Bordoloi Regional Institute of Mental Health Tezpur Assam India; ^3^ NIRMAAN Rehabilitation Facility Guwahati Assam India; ^4^ MIND India, Institute of Positive Mental Health and Research Guwahati Assam India; ^5^ School of Psychology, Lifton Terrace University of Leeds Leeds UK

**Keywords:** community education, LMIC, mental health, visual methods, youth substance abuse

## Abstract

**Introduction:**

Our aim is to evaluate the visually informed community mental health education materials cocreated in our research on youth substance misuse in Assam, India, and to reflect on what we might learn for similar initiatives in low‐ and middle‐income countries.

**Methods:**

Materials consist of: (i) images participants brought to the interview; (ii) 30 posters cocreated by participants to convey key messages from their interview; (iii) six short films on the implications of addiction, and (iv) an animation of our Pathways to Recovery model. We also created a community education package that incorporated these materials. We analyse feedback from three groups of events and a social media campaign, which drew variably across our materials and engaged a range of audiences.

**Results:**

Outcomes indicate the cocreation process and focus on the visual was successful in promoting young people's voice, increasing awareness and has potential for stigma reduction. Our educational package was deemed useful in increasing awareness and has potential for prevention and treatment.

**Conclusions:**

Our case study offers insights into community mental health education in low‐ and middle‐income countries, confirming the importance of cocreation, the usefulness of visual materials and the potential of social media campaigns while acknowledging the importance of local context in health messaging, particularly for stigmatized topics.

**Patient or Public Contribution:**

Service users were involved in the cocreation of the materials evaluated in this study and contributed as presenters in one of the events reported. Members of the public took part in events in which the materials were shared and provided us with the feedback analysed in this article.

## 1 INTRODUCTION

Mental health aligns most directly with United Nations (UN) Sustainable Development Goal 3 ‘Good Health and Well‐Being’, which aims by 2030 to reduce by one‐third premature mortality from noncommunicable diseases through prevention and treatment and promotion of mental health and well‐being.^1^ Mental health is one of the leading causes of ill‐health and disproportionately affects low‐ and middle‐income countries (LMIC)^2^ where the workforce gap is also large.^3^ We present a project based in Assam, India, in which we cocreated with participants visually informed community mental health education materials and reflect on what we might learn for similar initiatives in LMIC.

Mental health is framed increasingly as a crosscutting development issue. The *Lancet Commission on Global Mental Health and Sustainable Development* ^2^ argues that priorities should include: extending the mental health agenda to the general population; engaging a wide range of stakeholders beyond health; targeting social and environmental causes and making innovative use of nonspecialists and digital technologies. This direction is reflected also in the updated World Health Organization *Mental Health Action Plan 2013–2020*, which notes a positive shift in focus towards providing mental health care in the community rather than in institutional settings.^4^ At the same time, the *International Study of Discrimination and Stigma Outcomes Research Network on Mental Health* reports that, while there is cross‐cultural variation, mental health stigma and discrimination are universal but, importantly, also reversible.^5^

Participatory arts methods are burgeoning in global development research and practice.^6^ Such methods share a focus on working with concrete things together. ‘Doing with’ and not ‘doing to’ is an essential principle in development work alongside a privileging of local skills, knowledge and problem‐solving. Participatory arts are also well‐suited to work in LMIC given their inclusiveness and proven capacity to engage marginalized groups irrespective of educational level, language or income,^7^ facility to integrate local traditions and potential for innovation through digital technologies,^8^ as recommended by the *Lancet Commission*. Participatory arts overlap strongly with visually informed methods in which the spectacle produced, be it an image, object or activity, is central to securing engagement and impact.

The community mental health education materials examined in this article were developed as part of a research project on youth substance misuse in Assam. Assam is a state in northeast India in rapid, although uneven, modernization. It is recognized by the Northeast Council^9^ that the needs of women, children and youth require urgent attention. Moreover, the *Assam State Report of the National Mental Health Survey* ^10^ notes adolescent substance misuse as an important public health problem and recommends working closely with rehabilitation services, decreasing the stigma around substance misuse and encouraging help‐seeking through better public awareness.

A large survey by Singh et al.^11^ ^(p.2)^ confirms that mental health stigma is a chronic problem in India. The authors recommend ‘community education and training at a grassroots level’ and highlight the success of outreach projects. For example, Shahani^12^ ^(p.326)^ describes an ‘*Initiatives of Change’* community education programme undertaken in association with a high school in Mumbai: ‘Conducting workshop for parents made the male members of the family give up smoking, drinking and many of the addictions. Volunteers were assigned so as to see that recurrence of the same addiction does not take place’. Many private rehabilitation facilities also provide community outreach, involving their service users in activities as a valuable component of their recovery journey.

Our research focused on two groups of young Assamese people: 15–18‐year‐olds (seven males and eight females) who have resisted substance misuse (irrespective of tobacco use) despite being at increased risk due to this being a familial and/or peer issue; 19–24‐year‐olds (11 males and 4 females) who have engaged successfully with rehabilitation and demonstrated sobriety for at least 1 year.^13^ Two of our original research objectives are relevant to the current article: (i) to promote young Assamese people's voice with respect to substance use to reduce stigma and raise public awareness; (ii) to enhance local approaches to the prevention and treatment of youth substance use through visual materials. The aim of the present article is to evaluate the visually informed community mental health education materials we cocreated with our participants and to reflect on what we might learn from this case study more widely for similar initiatives in LMIC.

## 2 COCREATED VISUALLY INFORMED MATERIALS

Approval was obtained from the Ethics Committee of the LGB Regional Institute of Mental Health (LGBRIMH), Tezpur, India and from the Ethics Committee of the School of Psychology, University of Leeds, UK.

### 2.1 Images

Each participant was invited to take or collect a minimum of seven photographs or other images to assist convey their experiences of resisting or recovering from substance misuse.^7^ Detailed guidance was provided about image suitability. Participants brought between 7 and 34 images to share during the interview. Some portrayed life events while others represented thoughts, feelings and relationships.

### 2.2 Posters

Each participant was invited to design a poster to convey a message based on their personal experiences as shared during their photo‐led interview. All except one from the older group did so. A total of 30 posters were created, one from the younger group contributing two. Support was usually requested from the Research Fellow (RF), although each participant was always consulted and confirmed satisfaction with the final product. Posters conveyed a variety of messages around encouraging young people to avoid addictive substances and inviting constructive support and understanding from the general public.

### 2.3 Films

Each participant was invited to take part in training to enable them to cocreate a short film on the implications of substance addiction.^6^ Training was responsive to participant availability and involved storyboarding, using the equipment and editing, and the RF provided support throughout the process. Ten participants took part in film‐making: two from the younger and eight from the older group, alongside 25 of their peers. Six films were cocreated and ranged 5–17 min. The films conveyed messages about resisting substance use, destigmatising addiction and encouraging support for recovery (Table 1).

**Table 1:** Summary information on the cocreated films and animation

Order made	Group and film title	Cocreators		Length (mins)	Description
		Interviewees/peers			
19–24‐year‐olds in recovery (total *N* = 11 male, 4 female)					
1	*Diary of a Recovering Drug Addict*	1 Male	0	10	In four chapters of a recovery addict's life, this film highlights thoughts, feelings and experiences he had through his time from addiction to recovery.
2	*One for the Other*	3 Male	12 Male, 1 female	10	A young man and his family, ridiculed for his addiction, lend a helping hand to the same people who had pointed fingers at them.
3	*A Different Path to Recovery*	1 Female	0	5	As she walks to work, a young woman recalls how her path to recovery seemed different from that of men given the social expectations on her.
5	*Wrestling Against all Odds*	2 Male	0	8	A glimpse into the challenges one young man experiences trying to stay in recovery from addiction during CV‐19 lockdown.
6	*Ek Notun Probhat (A New Dawn)*	1 Female	7 Male, 5 female	17	This film in Assamese depicts the life problems of a young woman who, at the same time, struggles with substance addiction.
15–18‐year‐old at increased risk (total *N* = 7 male, 8 female)					
4	*Taint in the Lush Green*	2 Female	0	6	Based on a story told by two young women living in a small village in Assam. They make a plea for social change so that rural communities can receive help for substance addiction.

### 2.4 Animation

Analysis of the research interviews with the participants in recovery led to the development of a *Pathways to Recovery* model. The final model incorporated feedback from mental health and rehabilitation professionals in Assam and from two of our female and two of our male research participants.^13^ To obtain feedback, the RF drew a picture of the model as a looping series of pathways and staging‐post destinations. This proved an understandable and attractive format, and we had the idea to create a simple animation of a car driving along the pathways to communicate the model. We added a voice‐over conveying typical thoughts and feelings at the different stages selected from across the interviews. Four versions were produced: two in Assamese (male and female voice) and two in English (male and female voice).

### 2.5 Education package

**Image 1:**
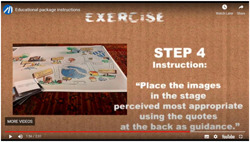
Still from the education package stop‐motion guidance animation.

We developed a community education package based on our model. The package consists of our animation, printable versions of the model in the illustrated form (with and without stage labels), six printable stage labels and 12 printable anonymized images (two per stage) brought to interview for which we had consent for public release. Each image was linked to an anonymized quote from an interview which illustrates a stage depicted in the model. The package includes also guidance notes explaining the model for the facilitator and suggesting a two‐part interactive session in which attendees are shown the animation and invited to place the stage labels and image/quotes in the right place on the unlabelled version of the model. A stop‐motion guidance animation demonstrates the process. The workshop format is intended to catalyse discussion of ways into and out of addiction and the sharing of personal experiences as deemed appropriate by the facilitator (Image 1, 1).

## 3 FEEDBACK ON MATERIALS

We undertook several knowledge exchange events in different formats for different stakeholders, drawing variably on our visual materials. We describe the three groups of events, the feedback received and the outcomes of the social media campaign we conducted towards the end of the project (Table 2).

**Table 2:** Event, audience demographic and visual materials evaluated

Event audience		Images (only)	Posters	Film(s)	Animation (only)	Education package (images and animation)
1	High school students		√	✓		
	General public		✓	✓		
2	PG mental health trainees			✓		✓
3	(Semi‐)rural women			✓	✓	
4	General public: youth, parents, community leaders, policy makers	✓	✓	✓		✓

Abbreviation: PG, postgraduate.

### 3.1 Event 1 (February 2021): Three community education workshops for high school students and the general public

**Image 2:**
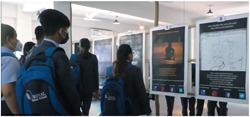
Poster exhibition at *Voice of the Youth: Resistance and Recovery from Addiction*.

In collaboration with our POs, we ran three community education workshops in the largest conurbation of Assam under the title *Voice of the Youth: Resistance and Recovery from Addiction*. We approached two local secondary schools, inviting students aged 15–18 years to attend Days 1 and 2. One school caters for a relatively affluent demographic (*N* = 44; Grade 11), the other for less affluent (*N* = 9: Grades 9 and 10). A total of 53 high school students accompanied by four staff attended over these 2 days (Image 2). The third day was advertised to the general public, with 44 people attending including some health professionals.

All 30 posters were exhibited and, on Days 1 and 2, attendees watched three films: *Diary of a Recovering Drug Addict*, *Ek Notun Probhat* and *A Different Path to Recovery*. The first two films were followed by a short talk by the participant who had cocreated the film and attendees were encouraged to ask questions. All six films were screened on Day 3. PO representatives and the RF also provided short talks about the project and interacted with attendees. Paper feedback sheets were handed out towards the end of the event and were completed by all 53 school students and by 31 members of the public.

#### 3.1.1 Poster feedback

Eight high school students gave verbal consent to a short impromptu interview with a company we hired to video‐record the event. These interviews were undertaken while the students were viewing the poster exhibit. Their responses covered the following themes: message relatability, bigger picture, spread the word, importance of family support and follow passion not drugs. In the formal feedback, attendees were invited to identify their favourite poster and to provide a short reason for their choice. They then responded to four questions about the impact of the posters with a Likert scale response (Tables 3 and 4).

**Table 3:** Favourite posters of the high school students and reason themes (*N* = 53)

Poster	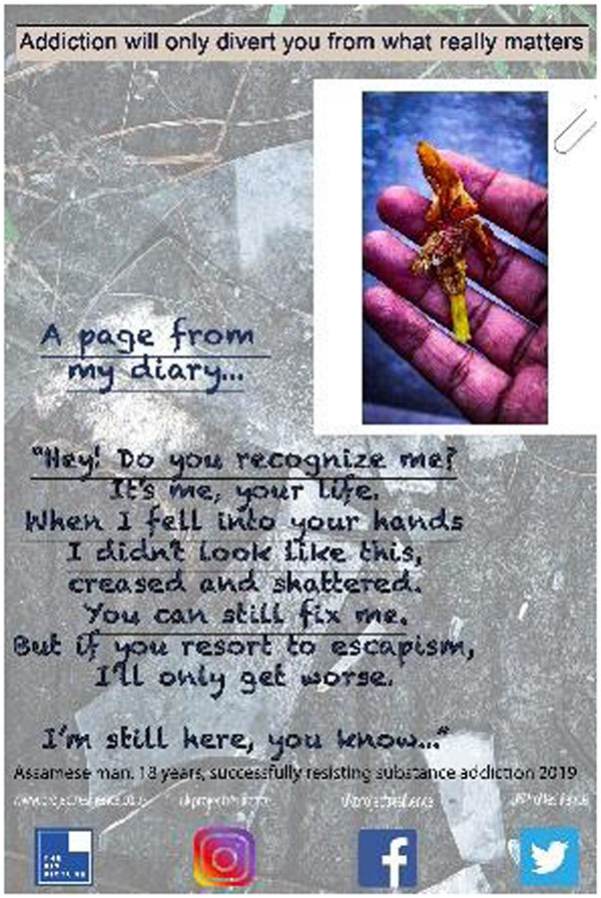	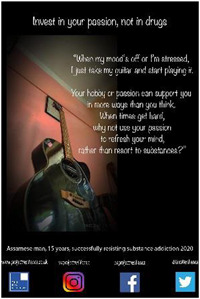	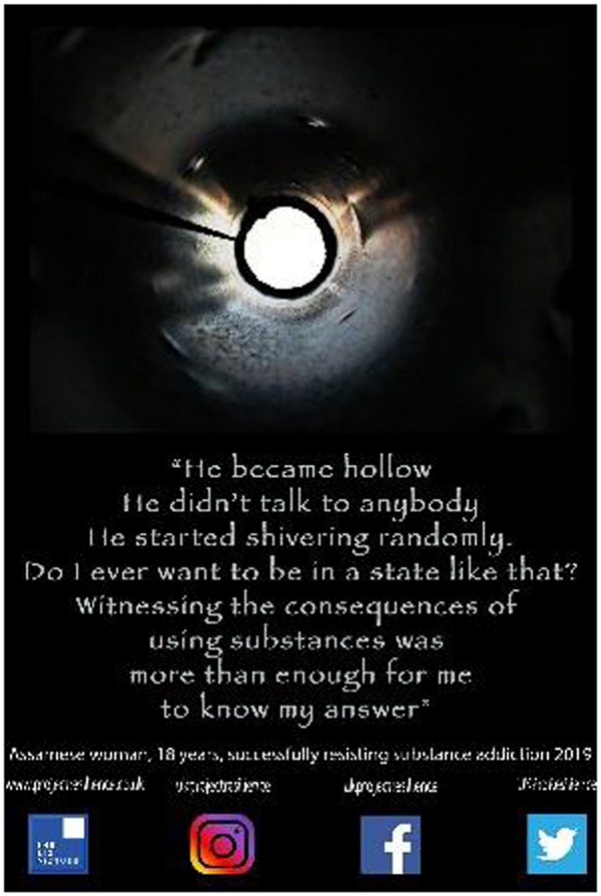
Participant	Male, 18 years	Male, 15 years	Female, 18 years
Votes	13	10	10
Themes	Importance of life itself	Highlights different path	Knowing consequences
	‘Thinking better’	Gives a solution	‘Simple yet beautiful’
	Message of hope	Focus on passion	
	Powerful image		

**Table 4:** Favourite posters of the general public and reason themes (*N* = 31)

Poster	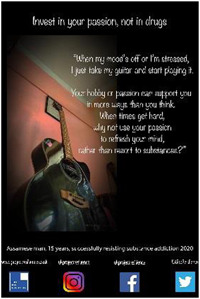	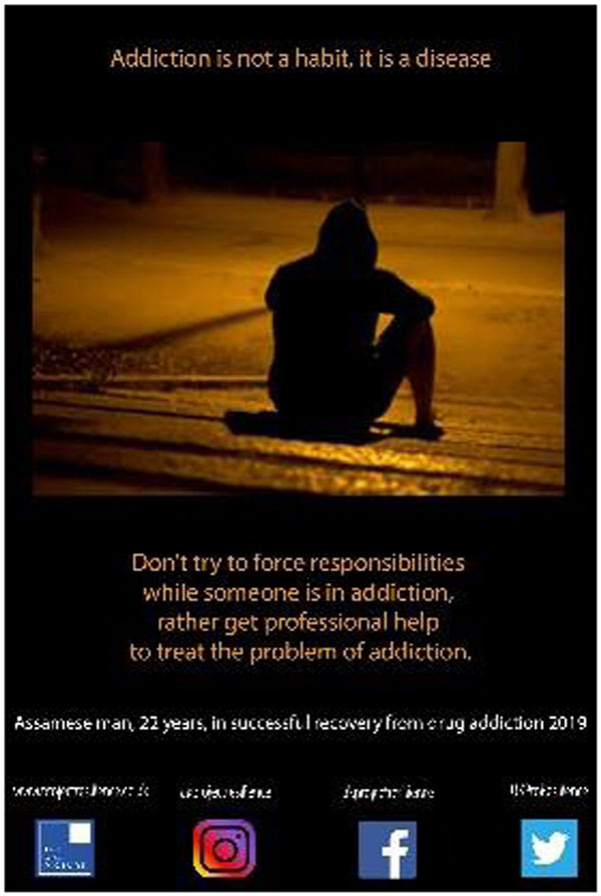	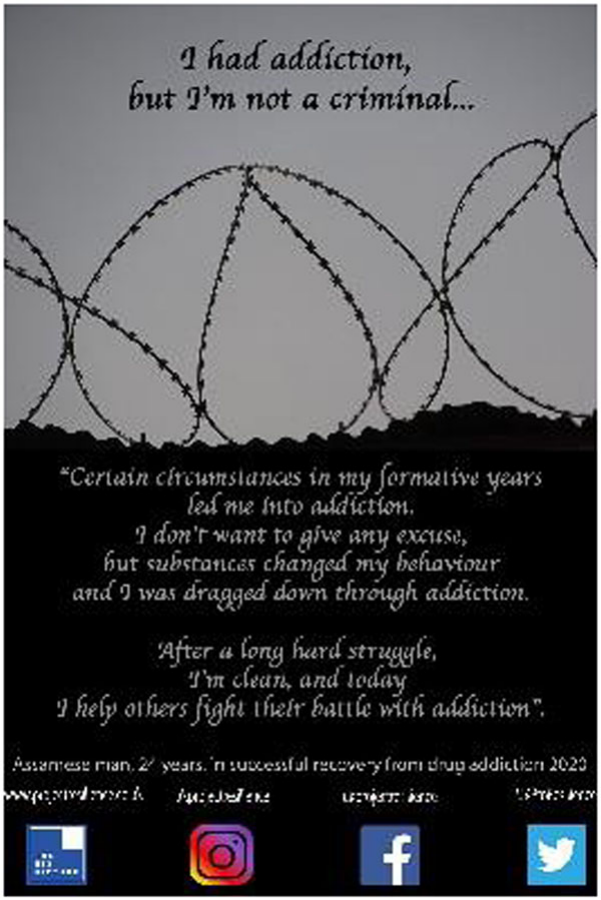	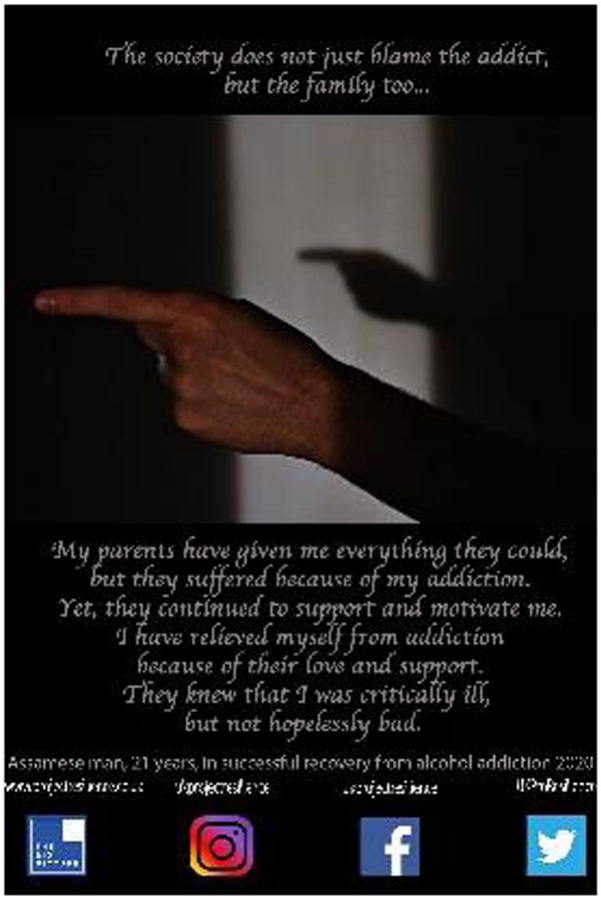
Participant	Male, 15 years	Male, 22 years	Male, 24 years	Male, 21 years
Votes	5	5	4	4
Themes	Shows a different path	Understanding an addict	Removes stigma	Faith in recovery
	Focus on passion	Provides facts	Can be overcome	Shows a family's pain
				Reality of a drug addict

All the most popular posters of the high school students were from the younger group of participants. This suggests that the message of resisting substances resonated particularly strongly with the high school students, and being ‘relatable’ was an important feature of the posters mentioned in the impromptu interviews. Reasons for liking the posters echoed this in that the students were inspired to consider a range of reasons to stay clean. This was reflected also in their impromptu interviews in which they also commented on the importance of support from family and community education. On the other hand, most of the posters popular with the general public were from the older group in recovery. Reasons were that they showed the wider impact of substance misuse, provided understanding and gave hope. One poster was rated highly by both high school students and the general public. Feedback reveals this poster was popular because it conveyed both a passionate message to resist substances and offered an alternative coping strategy.

Quantitative feedback was very strong from both students and the general public that the posters sent important messages about the problem of substance misuse (Table 5). Both groups were also optimistic that the posters could motivate young people to resist substances and could help young people in addiction have hope of recovery. However, although the students thought the posters could help adults understand more about problems associated with addictive substances for young people, the general public was less convinced.

**Table 5:** Response to the feedback statements on the posters from high school students (HS, *N* = 53) and the general public (GP, *N* = 31)

Feedback statements	Definitely		Somewhat		Not sure		Not so much		Not at all	
	HS	GP	HS	GP	HS	GP	HS	GP	HS	GP
The posters sent important messages about substance use	96%	93%	4%	7%	–		–		–	
The posters could motivate young people to resist substance use	72%	74%	26%	19%	2%	–	–		–	
The posters could help adults to understand more about the challenges of substance use for young people	77%	55%	19%	35%	4%	–	–	3%	–	
The posters could help young people in addiction have hope to recover	70%	71%	28%	19%	2%	6%	–		–	

*Note*: One response for GP missing for the first and fourth statement each; two responses for GP missing for the second statement.

#### 3.1.2 Film feedback

Feedback on the films was gathered through five questions inviting a free‐form response (Table 6). One of the main messages students took from the films is the potential for substance addiction to have a devastating effect. It was through seeing the possible long‐term negative impacts that they realized they should always find other solutions to their problems. Furthermore, some who used to judge and avoid people who used substances felt a new desire to help their peers to quit. Most students considered the films to have educational potential in guiding adults away from punitive reactions and towards helping young people to make positive life decisions.

**Table 6:** Feedback themes on the films from high school students (*N* = 53) and the general public (*N* = 31)

Questions	Feedback themes	
	High school students	General public
What key messages about substance use did you get from these films?	Ruin life	Recovery is possible
	Every problem has a solution	Ills of substance use
		Not judge
In what ways have the films changed your understanding of young people who use substances?	Understanding and helping	Understanding and helping
	Recovery is possible	Do not stigmatize
What impact could these films have on young people who see peers already taking substances?	Make peers understand	Motivate to understand
	Refrain from peer pressure	Motivate to recover
What impact could these films have on adults who know little about the challenges of substance use for young people?	Educate the adults	More informed
	Guide youth	Not judge but help
Is there anything else you would like to share with us?	Learned a lot	Carry the movement forward

One of the main messages that the general public took from the films is the possibility of recovery and challenging the stigma associated with substance addiction. The films encouraged them to embrace the importance of understanding and empathizing with young people suffering from addiction and to provide support towards the goal of recovery rather than judge them harshly. A few suggested carrying the work forward to allow more people─young and older, in rural and urban settings─to have the opportunity to view the films.

Both the students and the general public indicated that the films had helped them learn about the negative consequences that can befall many people, especially family members, as a result of one person's substance misuse. This appeared to be a key motivator identified by the students to resist getting involved in drugs and for the general public to make an effort to understand more about addiction and how to provide constructive support to those impacted.

### 3.2 Event 2 (August 2021): Workshop for postgraduate mental health trainees

**Image 3:**
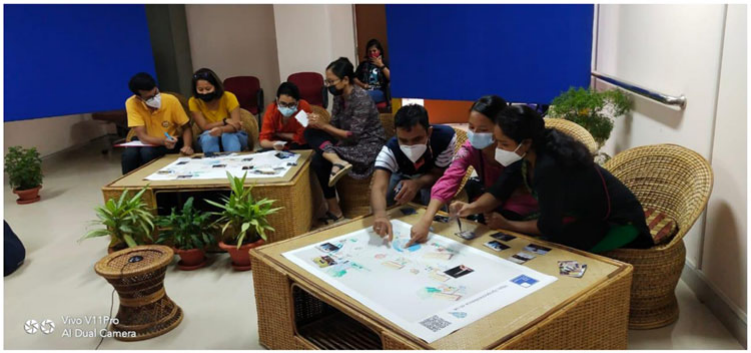
Postgraduate mental health trainee workshop at LGBRIHM.

We held a workshop at LGBRIHM (Image 3). This is one of the oldest mental health care institutes in India and advises the Assam State Government on relevant issues. LGBRIMH provides free inpatient, outpatient and community out‐research mental health services and postgraduate (PG) training programmes. Ten PG students aged 25–30 years attended, their studies ranging clinical psychology, psychiatry and psychiatric nursing.

The aim of the workshop was to assess the value of our education package and obtain advice on improvements. It was facilitated in person by our Project Advisor at LGBRIMH and by the RF online. The workshop commenced with a brief overview of the project and a screening of the *Pathways to Recovery* animation. Attendees were then divided into three groups and invited to place the image/quotes postcards on a large print of the pathways map next to the stage best illustrated. The facilitator discussed the placements with each group and used this to help explain the pathways to recovery model. The film *Taint in the Lush Green* was then screened, and attendees were invited to complete a feedback form.

#### 3.2.1 Workshop feedback

We asked three questions about the impact of the workshop with a Likert scale response (Table 7) and a further two questions inviting a free‐form response (Table 8): The first gathered feedback to help us improve the workshop and the second sought new ideas about how our *Pathways to Recovery* animation might be of use to practitioners.

**Table 7:** Response to the feedback statements on the workshop from PG trainees in mental health (*N* = 10)

Feedback statements	Definitely	Somewhat	Not sure	Not so much	Not at all
The workshop raised my awareness about youth substance use disorder	100%	–	–	–	–
The animation helped me understand different pathways from addiction to recovery	50%	40%	10%	–	–
The group activity helped deepen my understanding of the pathways to recovery	60%	30%	10%	–	–

Abbreviation: PG, postgraduate.

**Table 8:** Feedback themes on the workshop from PG trainees in mental health (*N* = 10)

Questions	Feedback themes
What worked well in the workshop and what could be improved?	Vivid and innovative
	Awareness better spread through visuals
	Quotes need more clarity
	More guidance at the start
How might the animation be used by service providers in the helping professions?	Simplifying complexity
	Nonthreatening
	Potential in group therapy
	Makes issues more realistic
	Hope for recovery

Abbreviation: PG, postgraduate.

Feedback on the education package was good. All attendees were very positive that the workshop raised their awareness about youth substance use disorder. With regard to the animation, 90% were reasonably positive that the animation helped them understand different pathways from addiction to recovery. Finally, 90% were reasonably positive that the group activity helped deepen their understanding of the pathways to recovery.

The qualitative feedback helped us improve our education package. We did so by adding an activity which allowed attendees to engage more deeply with the *Pathways to Recovery* animation, to understand better the stages of addiction illustrated and the trajectories between them. Hence, we added a printable map of the pathways without stage labels and printable stage labels. Our guidance now suggests that, after screening the animation, attendees are invited to place the stage labels in the right place on this map. This activity can be followed by a discussion about the stages and relationships between them and a second screening of the animation. We believe this will facilitate attendees to have a better understanding of the model before attempting to place the image/quote postcards against the appropriate stage.

### 3.3 Event 3 (September–October 2021): Four online community education events for women in rural and semi‐rural Assam

**Image 4:**
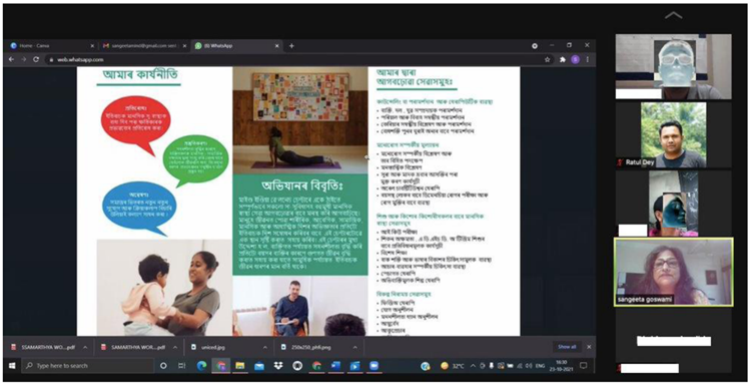
Screenshot from one online workshop with women's SHGs. SHG, Self Help Group.

Four online events were undertaken titled *Awareness on Substance Use Disorder and Alcohol Misuse to Enhance Community Wellbeing* (Table 9). These were conducted by our POs in association with Self Help Groups (SHGs) for women in three districts of Assam (Image 4). SHG is an initiative of the Assam Rural Development Society, the objective of which is to extend assistance to women of the tea tribes' community to enhance their quality of life and that of their families (https://www.ardsassam.com/self-help-groups/). The online workshops utilized our animation and one of our short films, *Diary of a Recovering Drug Addict*, to enhance awareness of substance misuse. The workshops also include information on referral services for addiction and advice about positive well‐being.

**Table 9:** Number of attendees and SHGs represented at online community education events by district

District of Assam	SHGs	Attendees
Golaghat	3	30
Golaghat	3	32
Jorhat	4	20
Goalpara	3	22
*Total*	*13*	*104*

Abbreviation: SHG, Self Help Group.

#### 3.3.1 Workshop feedback

Workshop feedback was designed not only to provide information to our POs about the women's psychosocial support and counselling needs but also to provide feedback on our materials. Feedback was provided by all attendees (total *N* = 104; Table 10).

**Table 10:** Quantitative feedback on the workshops for SHGs (*N* = 104)

Questions	**Excellent**	**Very good**	**Good**	**Average**
Coverage of the programme	–	91.35%	7.69%	0.96%
Rating of overall programme	66.34%	24.03%	8.65%	0.96%

	**Highly participatory**		**Participatory**	**Not participatory**
Training method	89.42%		10.57%	–

	**Yes**		**No**	**Don't know**
Do you find any topic irrelevant?	–		100%	–
Would you like to have a similar training programme in future?	94.23%		5.77%	n/a
Any new topic that needs to be included in the programme?	54.81%		19.23%	17.31%

Abbreviation: SHG, Self Help Group.

The workshops were rated highly by attendees in terms of overall quality and content covered, none of which was deemed irrelevant, and as highly participatory. The vast majority wanted similar training programmes in the future and over half indicated more topics on which they needed information. Of the qualitative feedback, Table 11 lists themes most relevant to our materials.

**Table 11:** Relevant feedback themes from the SHGs

Questions	Themes relevant to substance misuse
Any new topic that needs to be included in the programme?	Consequences on others
	Mobile addiction/cyber abuse
	Steps to support
	New and attractive activities to spread awareness
	Local language reading materials
Suggestions and comments	Need for such programmes in villages
	Community‐level programmes
	Face‐to‐face discussion needed
	Need training for youths and mothers
	Interesting video
	Need more short videos
	Target educational institutes
	TAlso, target male members of the family
Anything new that you have learned in the workshop?	Warning signs of substance abuse
	Treatment process
	Process of helping to recover
	Different types of substance abuse
	Reasons the young use substances
	Recovery pathways
	How to resist
	Role of parents
	Identify early users and prevent
	Impact of substance use

Abbreviation: SHG, Self Help Group.

Attendees demonstrated an appetite for more information on different types of addiction, the impact on others, and how to support people to avoid addictions and to stay in recovery. They indicated the need for greater addiction awareness at the community level in villages, schools and colleges and for young people, men and mothers, to employ engaging face‐to‐face methods using local languages, and enthusiasm for short videos. The latter was observed also by workshop leaders who note that the animation had the greatest impact on catalysing discussion. Learning included a greater understanding of substance misuse, the warning signs, prevention strategies, impact, treatment process and how to support recovery, including the role of parents.

### 3.4 Event 4: Social media campaign (January–February 2022)

**Image 5:**
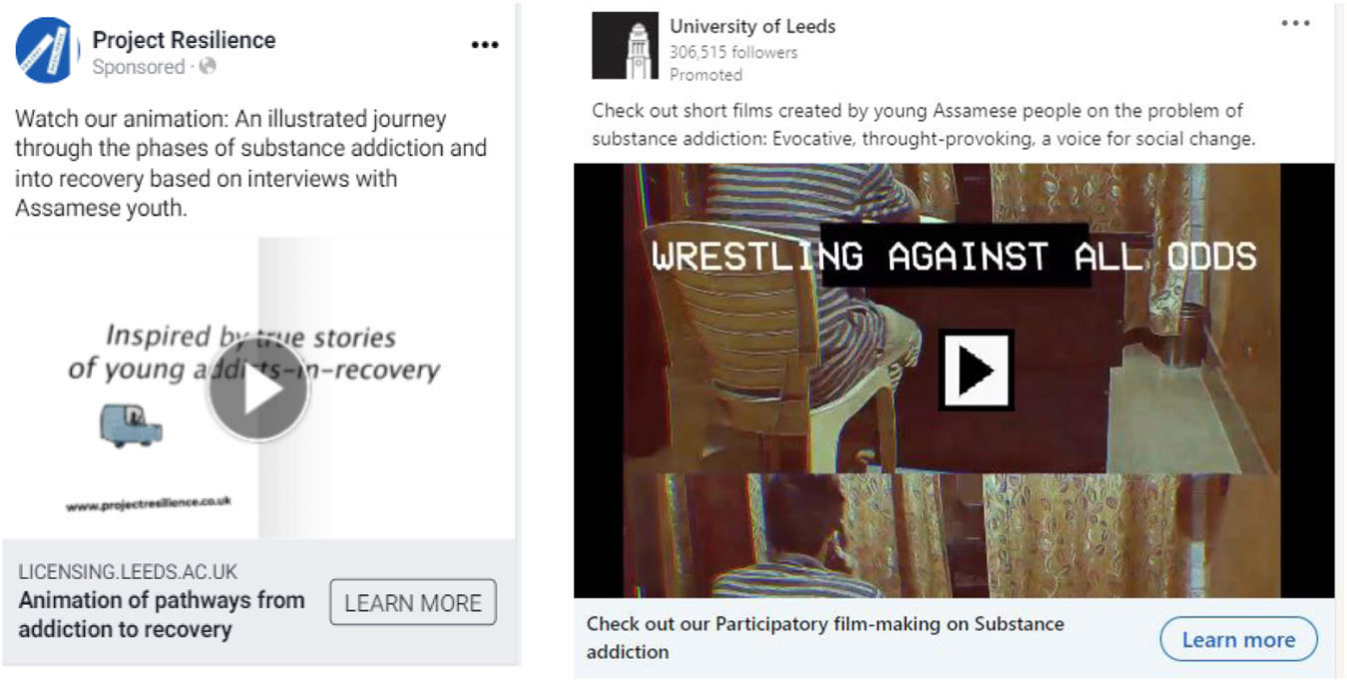
Example stills for Facebook and Linkedin short video adverts.

In the last 5 weeks of the project, we commissioned a social media campaign across four platforms: Facebook, Linkedin, Twitter and Instagram. We created seven short video adverts to highlight key aspects of our work, including four for the different visual materials produced, that is, images, posters, films and our education package (including images and animation) (Image 5). The other three adverts provided an overview of the project, policy recommendations and case studies. Each platform was given a campaign brief (Table 12) and employed its algorithms to identify accounts on which to place our promoted/sponsored posts. Each advert incorporated a ‘learn more’ button to our project webpage, except that for our animation/education package advert, which clicked through to our free intellectual property download page.

**Table 12:** Social media campaign target audiences plus brief

Region	Description	Age (years)	Education level	Interests
Assam	Youth	15–25	Secondary school, further education	Youth pop culture
Assam	Parents of teenagers	35–60	Basic to further education	Popular culture, traditional Indian culture, public affairs
Assam	Community leaders	40–70	Further education	Traditional Indian culture, public affairs
Assam, New Delhi, Bengaluru	Policy‐makers	30–70	Further education	Health policy, mental health, political and public affairs

**Figure 1:**
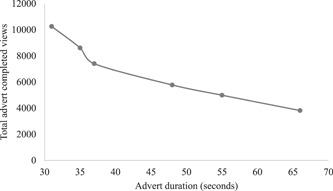
Social media advert duration against the total number of completed advert views. *Note: Point 1 represents two 31 s adverts with completed views of 10,256 and 10,253, respectively*.

We reviewed activity after 1 week and withdrew from Instagram because link clicks and video completions were not as strong as for the other platforms (Table 13). The most successful advert by total clicks, mean cost per click, and total engagements was for our images; the least successful for our posters. However, total video completions for the images advert was actually the lowest, while the films and animation/education package adverts had the joint highest number of completed views. In possible explanation, total video completions relate inversely to video duration and, at 55 s, our image advert was relatively long (Figure 1, 1). During and immediately following our campaign, there were 10 downloads of our education package, making a total to date of 17. The most successful platform by total clicks, mean cost per click and total engagements was Facebook; the least successful, excluding Instagram, was Linkedin. However, Facebook delivered the least number of video completions and Linkedin the most. Our adverts performed well above all‐industry benchmarks by cost per click, except for Twitter.

**Table 13:** Key outcome indicators by social media platform for our visual materials adverts

Indicators	Link clicks/cost per click				Engagement^a^/video completions				Total clicks/mean click cost	Total engagements/total video completions
Advert platform	Facebook	Linkedin	Twitter	*Instagram* b	Facebook	Linkedin	Twitter	*Instagram* b		
Images: Photo‐led interviews with young Assamese people 55 s	5752	115	865	*64*	53,327	122	292	*6686*	**6796**	**60,427**
	£0.04	£2.21	£0.31	*£0.40*	759	3248	933	*52*	**£0.12**	**4992**
Posters: Posters as messages about substance addiction 35 s	2309	116	805	*27*	24,112	124	311	*3293*	**3257**	**27,840**
	£0.05	£2.23	£0.36	*£1.51*	1067	5123	2354	*66*	**£0.22**	**8610**
Films: Short films about youth substance addiction 31 s	2536	95	712	*34*	41,476	99	342	*3652*	**3377**	**45,569**
	£0.10	£2.56	£0.37	*£0.51*	1724	5687	2767	*78*	**£0.24**	**10,256**
Education package/animation: Animation of pathways from addiction to recovery 31 s	3702	106	619	*5*	40,501	107	179	*488*	**4432**	**41,275**
	£0.08	£2.46	£0.42	*£0.81*	1539	6116	2581	*17*	**£0.19**	**10.253**
**Total clicks**|	**14,299**	**432**	**3001**	***130***	**159,416**	**452**	**1124**	***14,119***		
**Total engagements**										
**Mean click cost**|	**£0.07**	**£2.36**	**£0.37**	***£0.68***	**5089**	**20,174**	**8635**	***213***		
**Total video completions**	*£0.74* c	*£4.01* c	*£0.29* c	*£2.71* c						

*Note*: Bold values indicate that these are totals.

^a^ Likes, shares, comments and so forth.

^b^ Instagram 1 week, other platforms 5 weeks.

^c^ Average cost per click across industries.^14^

## 4 DISCUSSION

Our aim is to evaluate the visually informed community mental health education materials we cocreated with our research participants and to reflect on what we might learn from this case study more widely for similar initiatives in LMIC. We analysed feedback from three groups of events, which drew variably across our materials and engaged a range of relevant audiences. We also presented information allowing us to evaluate our social media campaign on several indicators.

### 4.1 Youth substance misuse in Assam

We exhibited the posters at the community education events for high school students and the general public, and the posters were featured in one of our social media adverts. Poster preference reflects Young's^15^ findings that source similarity, in terms of attitude and demographic, is an important influence on health message acceptance and underscores the benefits of cocreating materials with the target audience. Specifically, young people thought that the posters were an effective way to convey important messages about youth substance misuse, particularly when the message was relatable. On the other hand, the general public was enthused by posters that recognized the extended impact of addiction on the community and which offered hope. The merits of ‘hope’ versus ‘fear’ framing is a high‐profile debate in climate change messaging, Chapman et al.^16^ ^(p.850)^ concluding that ‘(e)motions should be viewed as one element of a broader, authentic communication strategy rather than as a magic bullet designed to trigger one response or another’. However, in the context of youth substance misuse, messaging hope for recovery seems an important factor in encouraging families and communities towards constructive, and away from punitive, reactions.

The general public was sceptical of the impact of the posters per se, and we speculate they might be most effective as part of a community action campaign, for example, to recruit peer‐to‐peer and parent‐to‐parent support. Moreover, the social media poster advert was the least successful of the four considered here. This was the case even though exhibition feedback suggested the posters had the social media ‘shareability’ dimensions of emotional intensity and self‐efficacy health messaging, such as alternative constructive coping strategies.^14^ However, the poster advert still achieved almost 28,000 engagements at only £0.22 per click, which is excellent performance against benchmarks.^17^

One or more of our films were screened during each of our events and were featured in one of our social media adverts. The films were deemed effective in conveying the long‐term, wide‐ranging negative consequences of substance misuse, spreading community awareness and inspiring the audience to take a constructive approach towards young people in addiction. In Assam, families often focus on scholastic achievement and material affluence, while young people can struggle due to social inequalities, poor opportunities to develop life skills and easy access to drugs.^18^ Communicating the *pervasive* destructive impacts of substance misuse may be an important drug prevention message for young people given that responsibility to family and community are central values in Assam.^19^ However, as noted above, this needs to be balanced with ‘hope‐messaging’, possibly encouraging a sense that restorative justice in terms of making amends and resurrecting social bonds is possible and constructive for all parties.^20^

Feedback conveyed appreciation that the films stemmed from real‐life experiences, again highlighting the value of our cocreative process. Quintero‐Johnson et al.^21^ ^(p.1121)^ advise caution in using testimonials in social media posts on stigmatized topics like mental health because they can ‘inadvertently provoke psychological resistance’. This underscores the sensitivity of mental health messaging. However, fortunately, supporting the general enthusiasm for our films and animation, these social media adverts were successful in attracting and holding interest, although we have no data on how they were interpreted.

We piloted our education package (including animation and images) with PG mental health trainees, the images from our photo‐led interviews were used in one of our social media adverts, and the advert featuring our animation directed people to our education package. Interestingly, the most successful advert, in general, was our images. However, its relative length did have a negative impact on total view completions and our advert performance on this criterion confirms an ideal length of around 30 s.^22^ The trainees considered the education package valuable in raising their awareness and promoting understanding of the pathways into, and out of, addiction. In relation to mental health social media posts, Swick et al.^23^ ^(p.2)^ state that ‘(a) challenge in providing community education is ensuring that the information is understandable and accessible. This requires the ability to transform complicated medical information into simplified terms’. Portraying our academic outcomes in a 4‐minute, metaphorical ‘pathways’ animation seems to have been successful in this regard, provoking workshop discussion, attracting excellent attention on social media and triggering downloads of the education materials.

We hereby summarize how our visually informed materials helped us achieve two of our original research objectives. First, the cocreation process and focus on visual materials did seem to be successful in promoting young Assamese people's voice with respect to substance misuse. We secured an excellent engagement with image collection and poster‐ and film‐making. Moreover, feedback from the events indicated increased awareness of the challenges young people face, the possibility of constructive approaches towards helping young people avoid and recover from substance misuse and, hence, indicates the potential at least for stigma reduction. Second, we have early indications that our educational package and associated *Pathways to Recovery* animation have potential in prevention and treatment and that visual materials have been an important aspect of the success of our project. Building on this study, we could evaluate the effectiveness of the posters alongside goal‐directed community education initiatives, trial the enhanced education package with a range of stakeholders and work closely with stakeholders to understand better the nuances of mental health messaging in Assam.

### 4.2 Community mental health education in LMIC

With respect to mental health challenges in LMIC, Shen et al.^4^(p.313) observed that ‘different countries have adapted deinstitutionalization in ways to meet idiosyncratic situations and population needs’ and that more needs to be done on implementation strategies. Hence, while much of what we have learned will be transferable to other LMIC, the context in terms of mental health foci, government policy and the audience is critical. However, commonalities will include working to reduce mental health stigma, embedding prevention initiatives in the community, and providing tools and strategies to help people support each other. Community education is a key aspect of this picture.

Our study confirms the importance of cocreating education material. This should involve a range of stakeholders who are affected differently by the ramifications of mental health issues, including parents, peers and community leaders. True cocreation facilitates choice about how to incorporate local knowledge, traditions and sensitivities into education materials and to make these available in local languages.^23^

Our study also confirms the potential of visually informed material to engage community interest and convey information. It is important that materials take into account local visual culture and customs so that the mental health message is communicated in an acceptable and effective way. This includes employing a range of visual modes, such as photographs, posters, film and animation to facilitate inclusivity, ownership and dissemination. Moreover, the act of cocreating visual material, itself, provides a forum in which people can reflect on their experiences of, and perspectives on, mental health and initiate meaningful conversations. This is in tune with the humanitarian and global challenges research sectors, which forefront partnership working and community‐led solutions.^6^

Finally, our cocreated visual materials helped us secure excellent community engagement in our social media campaign. Karthikeyan^24^ ^(p.527)^ notes that cheap mobile devices mean that ‘usage of social media among people of all ages, especially the youth and children are very high in developing countries like India’. Hence, it is likely that social media can be used successfully in many LMIC to reach out actively to a large audience who would otherwise be extremely difficult to engage. However, the interactions between mental health messages, social media platforms and audience are complicated and need to be considered at the planning stage.^21^

In conclusion, our case study project on youth substance misuse in Assam offers insights into the use of cocreated visually informed community mental health education in LMIC. As stated in the introduction, mental health is framed increasingly as a crosscutting development issue and our project has engaged with forward‐looking priorities, such as involving a wide range of stakeholders beyond health, targeting social causes and making innovative use of digital technologies.^2^ Integrating mental health community education within the larger global development agenda could also be helpful in broaching this largely stigmatized topic within the easier‐to‐have conversations.^25^

## AUTHOR CONTRIBUTIONS

**Raginie Duara**: Conceptualization (supporting); data curation (lead); formal analysis (lead); investigation (lead); methodology (supporting); project administration (supporting); visualization (equal); writing – original draft preparation (equal). **Diptarup Chowdhury**: Investigation (equal); resources (equal); validation (equal); writing – review and editing (equal). **Ratul Dey**: Investigation (equal); resources (equal); validation (equal); writing – review and editing (equal). **Sangeeta Goswami**: Conceptualization (supporting); investigation (equal); methodology (supporting); project administration (supporting); resources (equal); validation (equal); writing – review and editing (equal). **Anna Madill**: Conceptualization (lead); formal analysis (supporting); funding acquisition (lead); methodology (lead); project administration (lead); supervision (lead); visualization (equal); writing – original draft preparation (equal).

## CONFLICT OF INTEREST

The authors declare no conflict of interest.

## ETHICS STATEMENT

Approval was obtained from the Ethics Committee of the Lokopriya Gopinath Bordoloi Regional Institute of Mental Health, Assam and from the Ethics Committee of the School of Psychology, University of Leeds, UK. The study conforms to the Declaration of Helsinki guidelines. All persons gave their free informed consent before their inclusion in the study.

## Data Availability

The data that support the findings of this study are openly available in ReShare at https://reshare.ukdataservice.ac.uk/855418/, reference number 10.5255/UKDA‐SN‐855418.
